# Molecular triage on HPV-positive samples in a cervical screening setting

**DOI:** 10.1371/journal.pone.0333539

**Published:** 2025-10-13

**Authors:** Gabriella Lillsunde Larsson, Jessica Carlsson, Gisela Helenius, Lovisa Bergengren

**Affiliations:** 1 School of Health Sciences, Örebro University, Örebro, Sweden; 2 Department of Laboratory Medicine, Faculty of Medicine and Health, Örebro University, Örebro, Sweden; 3 Department of Urology, Faculty of Medicine and Health, Örebro University, Örebro, Sweden; 4 Department of Women’s Health, Faculty of Medicine and Health, Örebro University, Örebro, Sweden; Kirklareli Universitesi, TÜRKIYE

## Abstract

**Objective:**

To improve human papilloma virus (HPV) screening, more effective triage methods for HPV-positive samples need development and validation. Cytology, the most common triage method today, is subjective and can only be applied to professionally collected samples. Methylation status has been shown to be informative, as genes are highly methylated in HPV-induced cervical dysplasia and cancer. This study aimed to assess whether triaging HPV-positive samples using molecular methods, such as methylation and genotyping for high-risk HPV types, could be as effective as cytology in cervical screening.

**Methods:**

A retrospective biobank study was conducted on HPV-positive samples collected in 2017–2018, analyzing FAM19A4/MiR-124-2 hypermethylation and HPV genotyping for types 16, 18, 31, 33, 45, 52, and/or 59, comparing these results to cytology triage for detecting histologically confirmed high-grade squamous intraepithelial lesions (HSIL) and cancer.

**Results:**

Results from 1915 positive screening samples were analyzed, including 1052 follow-up biopsies with 402 HSIL or cancer cases. Genotyping showed slightly higher sensitivity than cytology but lower specificity, while methylation had higher specificity but much lower sensitivity. Cytology’s positive predictive value (PPV) was 36%, with lower PPVs for the molecular methods. Combining molecular methods increased the PPV but significantly reduced sensitivity.

**Conclusions:**

Based on these findings with molecular methods reducing sensitivity, we do not recommend adopting the molecular triage methods evaluated in this study in the Swedish setting. The trade-off between sensitivity and specificity does not support a change from the current cytology-based triage approach.

## Introduction

Estimations predict that worldwide almost 730,000 women will develop cervical cancer by 2030, and this trend will not decline until there are more well-organized screening and vaccination programs with high participation rates [1]. Since this is not the case in many countries, cervical cancer control will rely primarily on the prevention of cancer after women have been exposed to human papilloma virus (HPV), the cause of cervical cancer, for many years to come.

Cervical screening today is HPV-based in many countries, and a key challenge is managing women who test positive for high-risk HPV (hrHPV). There is convincing evidence that HPV screening detects all types of dysplasia and cervical cancer to a greater extent compared to cytology screening [2–4]. However, there is also compelling evidence that HPV screening leads to increased clinical follow-up demands due to a higher rate of false positives cases [5–6]. HPV is a sensitive test, and even with cytology as a triage, many HPV-positive women are unnecessarily clinically examined, since many HPV infections clear over time and dysplasia may regress spontaneously [7]. This is burdensome for healthcare systems in terms of both workload and costs, and also creates a lot of anxiety for women [8]. In the Region of Örebro County and across Sweden, women with abnormal cytology results as part of triage are referred to colposcopy within 2–3 months. Women who test positive for HPV but have normal cytology are invited for a follow-up test after 18–36 months, depending on the HPV type. If the follow-up test also is positive for the same HPV genotype, the woman is also referred to colposcopy [9]. During colposcopy, biopsies are taken based on the appearance of the cervix, along with the results of the HPV test and cytology.

To enhance the effectiveness of HPV screening, further development and validation of triage methods for hrHPV-positive samples are essential. Cytology, the most widely used triage method today, is subjective and can only be applied to professionally collected samples. Vaginal self-sampling is becoming increasingly common in cervical screening due to its simplicity and inclusivity. However, with self-collected sampling there are no cells from the cervix to analyze cytologically, and the demand for new triage methods is evident. Methods to increase specificity following a positive HPV test include HPV genotyping, as different genotypes are linked to different risks of dysplasia and cancer development [10–12]. Furthermore, other biomarkers could potentially help differentiate between productive HPV infections associated with dysplastic lesions with a high regression rate and transforming HPV infections with a presumed higher risk of progression to cervical cancer [13]. For example, the hypermethylation of protective genes that typically counteract cellular changes could be informative. The hypermethylation status of the human genes FAM19A4 and miR-124-2 has been shown to be informative, since these genes are highly methylated in HPV-caused cervical dysplasia and cervical cancer [14,15].

Until recently, there have been limited data in the literature regarding molecular triage within an implemented screening program. This gap led to the current retrospective study, which performed methylation analysis and HPV genotyping on HPV-positive archived samples from 2017–2018. The study evaluated these molecular results against histologically verified high-grade squamous intraepithelial lesions (HSIL). The aim of this study was to assess whether molecular analysis methods alone for triaging HPV-positive samples were as effective as cytology in cervical screening.

## Materials and methods

### Study design

The study included women aged 30 years and older who participated in cervical cancer screening in the Region of Örebro County, Sweden, during 2017–2018. Cervical samples were collected by midwives using liquid-based sampling (Hologic, Marlborough, MA, USA) for primary HPV testing, which was introduced in the region in 2016. The HPV screening method used was HPV Aptima (Hologic, Marlborough, MA, USA), which detects mRNA from 14 hrHPV types (16/18/31/33/35/39/45/51/52/56/58/59/66/68). The study included women with a positive HPV screening test, though genotype distribution from the screening test was not reported.

A total of 1929 women tested positive with HPV Aptima and were included in the study. They were followed according to national screening guidelines at the time, which included cytology triage after a hrHPV-positive screening test. Residual material from the liquid-based cytology at the time of diagnosis was biobanked and used for further analysis in this study, including DNA extraction, HPV genotyping, and FAM19A4/MiR-124-2 hypermethylation status. All analyses were performed in parallel and without knowledge of the histological endpoint. The study compared cytology triage with HPV genotyping and FAM19A4/MiR-124-2 hypermethylation as triage methods. The endpoint was histologically confirmed HSIL or worse (HSIL+) from biopsies collected during screening follow-up between 2017 and 2022. In accordance with existing screening protocols, histological biopsies were performed based on cytology and examination results. As this study utilized real-world data, women who did not undergo histology were assumed to have a normal endpoint.

### Cytology triage and clinical follow- up

The screening algorithm in the Region of Örebro County in 2017–2018 included cytology analysis following an hrHPV-positive screening test. Findings were reported according to the Bethesda classification system, which includes atypical squamous cells of undetermined significance (ASCUS); atypical squamous cells, cannot exclude high-grade lesion (ASC-H); low-grade squamous intraepithelial lesion (LSIL); high-grade squamous intraepithelial lesion (HSIL); squamous cell carcinoma; atypical glandular cells; adenocarcinoma in situ; and adenocarcinoma [16]. Women with cytological findings of ASCUS or worse (ASCUS+) were referred to colposcopy and clinical follow-up, whereas women with normal cytology were invited to a new screening test in 3 years. Routinely histological biopsies or loop excisional biopsies were not performed on all women if the colposcopy examination was found normal.

Histological biopsies, loop excisional biopsies, or hysterectomy findings were formalin fixed, paraffin-embedded, sectioned, and stained with hematoxylin and eosin. The diagnostic procedures and criteria followed national and international guidelines, with clinical pathologists evaluating all cases within the normal screening setting. Findings were classified as LSIL, HSIL, or cancer, with HSIL and cancer outcomes defined as HSIL + , as Sweden has a routine not to distinguish between cervical intraepithelial neoplasia, CIN, 2 and 3. The primary outcome in the study setup was histological HSIL + , while histological LSIL, normal biopsy, and women without histological samples taken were considered as controls. The follow-up time for included women was a minimum of 3 years.

### HPV genotyping

Biobanked samples were used for DNA extraction. From a biobanked pellet prepared with the Freedom EVO platform (Tecan, Männdorf, Switzerland) according to Perskvist *et al*. [17], 100 µl was used for DNA extraction on the QIAcube (Qiagen, Hilden, Germany). A DNA-based test, the Anyplex II HPV28 (Seegene, Seoul, Korea) was used for HPV genotyping. The real-time PCR test detects 28 different genotypes, including the 14 genotypes in the screening mRNA test: HPV 6/11/16/18/26/31/33/35/39/40/42/43/44/45/51/52/53/54/56/58/59/61/66/68/69/70/73/82. A human control gene was also included in the test to verify cell content. The test provides individual genotyping results, including information on multiple infections. For a negative result, the human control gene had to yield a valid result.

In this study, the 14 hrHPV types were grouped into three categories according to Swedish national guidelines [9]. HPV 16, 18, and 45 are considered highly oncogenic (HONC); HPV 31, 33, 52, and 58 are considered moderately oncogenic (MONC); and HPV 35, 39, 51, 56, 59, 66, and 68 are considered low oncogenic (LONC). The highest oncogenic group determined in which positive genotype group the sample was included for the calculations.

### FAM19A4/miR-124-2 hypermethylation

DNA extraction was performed as described above, and the same extract was used for both genotyping and methylation when sufficient DNA was present. In some cases, an additional DNA extraction was necessary due to low DNA concentration in the previous extraction.

Extracted DNA was treated with bisulfite using the EZ DNA Methylation-Gold assay (Zymo Research, Irvine, CA, USA). Sample hypermethylation of FAM19A4/miR-124-2 was evaluated with the Qiasure test (Qiagen), which is a methylation-specific real-time PCR. This test provides individual results for the genes FAM19A4 and miR-124-2, and a sample is determined hypermethylation-positive if either or both targets are positive. Automatic interpretation is performed by Rotor-Gene Assay Manager software (Qiagen), where target genes are compared to an internal control gene and an internal calibrator consisting of a low-copy plasmid.

### Data and statistical analysis

Continuous variables were described using medians and interquartile range (IQR), while categorical variables were described with absolute and relative frequencies. Chi-square tests were used to test for associations between categorical variables. Sensitivity, specificity, negative predictive values (NPV), positive predictive values (PPV), and 95% confidence intervals (95% CIs) were calculated for each screening strategy, based on histological outcomes (HSIL+ compared to controls). All analyses were performed in SPSS version 22.0 (IBM SPSS, New York, NY, USA). A statistical significance was considered at *p* < 0.05. Sub-analyses were conducted to compare screening methods in women aged 30–40 years versus those aged 60–70 years.

### Ethics statement and data collection

The study was approved by the Regional Ethical Authority in Stockholm (approval number 2021–03050), need for consent was waived by the ethics committee. The study was conducted using existing samples stored in the biobank of Region Örebro County. All samples were labeled with a unique code. The code key was stored at the Department of Laboratory Medicine, Örebro University Hospital, and was accessible to the research team via a secure storage platform within Region Örebro County’s IT infrastructure, which requires dual authentication for access. All data were analyzed in a pseudonymized form. Data collected for research from archived samples started October 19, 2021.

## Results

A total of 1915 screening samples with hrHPV-positive mRNA from women aged 30–70 years, collected between 1 January 2017 and 31 December 2018, were included in the dataset. The median age of the women was 41 years (IQR = 16 years). Fourteen patients were excluded because their sample was missing from the biobank.

Histological outcomes in this material included 1052 biopsies, whereas in 863 positive screening cases no biopsies were sampled, most often due to normal cytological triage or normal colposcopy. This real-world study setup uses the current screening algorithm as a baseline for comparison with the investigated methods. In this algorithm, an HPV-positive screening result is followed by cytology and further examination, and depending on the cytology results and examination, a biopsy may be taken. Hence, the numbers of HSIL for all methodological comparisons are based on the results of cytology output, since neither HPV genotyping nor methylation rendered cytological and histological examinations. Of the 1052 histological samples, 402 (38.2%) were HSIL+ samples, including 19 cancer cases, while the remaining were normal or LSIL: 313 (29.7%) and 337 (32.0%), respectively (controls). Of the HSIL+ cases, 66 were detected at the next screening round (3 years +). Of these 57 were MONC and HONC positive in the screening test, 55 had normal cytology, nine had ASCUS/LSIL, one had HSIL and one sample had no cytological diagnosis at screening test. Methylation positivity was seen in 11 of these samples.

### Cytology

**Fig 1:**
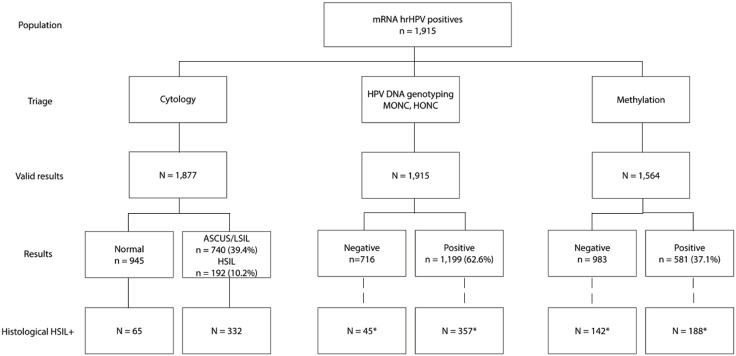
Flowchart of test results. The 402 histological HSIL+ cases are detected with one or more triage methods. Eighty-five HSIL+ cases were not positive for either of the triage tests.

Among the 1915 screening-positive samples, 945 had normal cytology (50.3%), 740 had ASCUS or LSIL (39.4%), and 192 had HSIL (10.2%) (Fig 1). Three samples were missing, and 35 did not have sufficient material to perform the cytological analysis. Among women aged 30–40 years, 54.1% had aberrant cytology compared to 37.6% among women 60–70 years of age (95% CI for difference: 0.09–0.24, p < 0.001).

Cytology as a triage for HPV-positive screening samples, detecting ASCUS + , yielded a PPV of 35.6% (95% CI: 33.9–37.4), where 332 out of 932 samples with atypia rendered an HSIL+ diagnosis, Table 1. Among cases with normal cytology, 65 HSIL+ cases (17%) were detected (Fig 1). Of these, 55 out of 65 were detected after the next screening round (3 years +).

**Table 1:** Sensitivity, specificity, positive predictive value (PPV), negative predictive value (NPV) and area under the curve (AUC) for histological HSIL+ of five triage strategies for hrHPV-positive women.

Triage test	Sensitivity (95% CI)	Specificity (95% CI)	NPV (95% CI)	PPV (95% CI)	AUC (95% CI)
Cytology	83.6 (79.6–87.1)	59.5 (56.9–62.0)	93.1 (91.5–94.4)	35.6 (33.9–37.4)	0.72 (0.69–0.74)
MONC & HONC genotypes	88.8 (85.3–91.7)	44.4 (41.8–46.9)	93.7 (91.8–95.2)	29.8 (28.6–31.0)	0.67 (0.64–0.69)
HONC genotype	56.2 (51.2–61.1)	70.7 (68.3–73.0)	85.9 (84.4–87.2)	33.8 (31.2–36.4)	0.64 (0.60–0.67)
Methylation	57.1 (51.6–62.6)	68.2 (65.5–70.8)	85.7 (84.0–87.2)	32.4 (29.7–35.1)	0.63 (0.59–0.66)
MONC & HONC genotypes and methylation	50.5 (44.9–55.8)	81.5 (79.2–83.6)	86.1 (84.7–87.4)	42.0 (38.2–45.9)	0.71 (0.68–0.74)
Cytology, MONC & HONC genotypes, and methylation	48.9 (43.4–54.5)	91.3 (89.6–92.9)	87.0 (85.7–88.1)	60.2 (55.0–65.2)	0.70 (0.67–0.74)

HSIL+ = high-grade squamous intraepithelial lesions and cancer, MONC = moderately oncogenic hrHPV types, HONC = highly oncogenic hrHPV types.

CI = Confidence interval.

### HPV genotyping

When analyzing for HPV DNA on the mRNA-positive screening samples, 6.4% of the samples were DNA-negative. Among the HPV DNA-positive samples, there were 669 samples classified as HONC, 530 samples as MONC, and 496 samples as LONC. The remaining DNA-positive samples contained genotypes outside of the defined risk groups but were included in the Anyplex II HPV28 analysis setup. There was a difference between age groups when comparing HPV genotype positivity: the older group of women aged 60–70 years had fewer HONC and MONC genotypes compared to the women 30–40 years (54.5% vs. 72.6%, respectively, 95% CI for % difference: 9.8–25.6, p < 0.0001).

Analyzing the results based on oncogenicity and histological outcomes, from the cytology triage pathway, the results showed a significantly higher proportion of HONC genotypes (57.8%) in histological HSIL+ compared to both MONC (33.5%) and LONC (8.7%) (p < 0.0001). HONC positives alone (226/669, 33.8%) had the best PPV among the tested molecular triage markers individually for detecting HSIL+ in histology, compared to MONC positives (131/530, 24.7%), LONC positives (398/1877, 21.2%) or a combination of MONC and HONC positives (357/1199, 29.8%).

### Methylation

A total of 351 samples had missing methylation results due to lack of material in the biobank, leaving 1564 samples in the analysis. Negative methylation status was observed in 63% of the 1564 positive screening samples where methylation analysis was performed. Hypermethylation positivity was seen in 188 of the 330 screening cases (57%) with HSIL+ biopsies. Among all the hypermethylation-positive samples, 32% had HSIL+ histologies (Fig 1). Of methylation-negative samples in triage, 142 out of 983 (14.4%) rendered a HSIL+ in histology.

When comparing age groups, for women between 60 to 70 years of age, 39.4% of the screening samples were methylation-positive, compared to 28.6% for women 30 to 40 years of age (p = 0.011, 95% CI for % difference: 2.3–19.7).

### Combined triage

When combining genotyping for MONC and HONC and methylation as triage, specificity was higher than each test separately, at 81.5 (95% CI: 79.18–83.59), while the sensitivity declined to 50.5 (95% CI: 44.92–55.99).

A combination of positivity from all three triage strategies tested on 1536 samples (cytology, genotyping MONC and HONC, and methylation), yielded a specificity of 91.3% (95% CI: 89.6–92.9) and a PPV of 60.2% (95% CI: 55.0–65.2), which is higher than the other triage strategies. However, sensitivity was lower compared to all other strategies (48.9%, 95% CI:43.4–54.5), with an NPV of 87.0% (95% CI: 85.7–88.1), Table 1.

The proportion of methylation-positives was higher in the HONC genotype group compared to the LONC genotype group, 43.4% versus 31.7%, (95% CI for % difference: 5.5–17.7, **p* *= 0.0002) as well as compared to the MONC group, 43.4% versus 37% (95% CI for % difference: 0.21–12.5, *p* = 0.043). Methylation did not differ between the MONC and LONC genotypes (95% CI for % difference: −1.1–11.6, *p* = 0.10).

## Discussion

In this retrospective study using HPV-positive screening samples collected in 2017–2018 in the Region of Örebro County, Sweden, we evaluated whether molecular testing with HPV genotyping and hypermethylation of FAM19A4/miR-124-2 are sufficient as triage tests instead of cytology. The results from this study indicate that using only molecular analysis methods is not as effective as cytology for triage in this cervical screening setting. The best option, as seen in this real-world data study, appears to be a combination of cytology with both molecular triage methods.

The baseline setup for this real-world triage study is that all included women were HPV-positive from an mRNA-based screening analysis. From this group of HPV-positive women, the specificity of the cytology triage was surprisingly low, at only 59.5%. Importantly, in our study group, we had a relatively large group of cytology ASCUS cases, thereby rendering clinical follow-up and consequently impacting the final outputs. This classification might differ between laboratories and studies. Additionally, knowing the HPV status before performing the cytology assessment can influence the subjective analysis, leading to overdiagnosis. Therefore, other methods are needed to increase specificity, with genotyping being one of them because different genotypes carry different risks of cancer [10].

Genotyping data from this study does not support the use of HONC genotypes alone as triage, nor HONC and MONC genotypes together. Despite an increased number of HSIL+ cases in HONC compared to MONC and LONC, neither of these triage options has both acceptable sensitivity and specificity. The current separation in genotype groups was used based on previous knowledge that women carrying HONC and MONC genotypes have the highest risk for developing HSIL + . However, it was also interesting to evaluate whether specificity could be retained by excluding the LONC genotypes in triage while including them in the screening test. When using HONC and MONC as triage for the HPV-positive women, sensitivity and NPV were high, while specificity was lower compared to cytology, which could yield an unacceptable number of false positives and consequently unnecessary use of health care and human resources.

The method used for genotyping is DNA-based, whereas the screening setup in Örebro uses an mRNA assay. Discrepancies between these methods have been shown before [18] and also for APTIMA compared to other methods besides Anyplex II HPV28 [19]. In this study, we report that 6.4% of mRNA-positive screening samples were HPV DNA negative. Preferably, a screening method using mRNA should address relevant HPV infections that need attention and not include DNA-negative transient ones. Some cross-reaction for low-risk genotypes in the APTIMA assay setup might explain these findings. However, it is not optimal to use two different methods for HPV detection and genotyping, especially if this leads to identifying women who are later HPV-negative, resulting in unnecessary clinical follow-up.

The sensitivity of FAM19A4/miR124-2 methylation for HSIL+ in the current study was 57.1% (95% CI: 51.6–62.6), which is considerably lower compared to both MONC/HONC genotyping and cytology. This aligns to some extent with results from a multicenter study including four European countries [20], where sensitivity for moderate cervical squamous intraepithelial neoplasia, CIN2, varied between 33% and 61%, and for severe cervical squamous intraepithelial neoplasia, CIN3, overall sensitivity was 77%. The same article showed higher sensitivity for cervical cancer, 95%, of which we had too few cases to analyze in this study (n = 19).

Additionally, comparing the studies is difficult, since, in the Swedish study setting, data are from the screening setup where HSIL results are not further divided into CIN2 and CIN3. Therefore, in a national context for Sweden, the focus should be on finding a triage method sufficient to include HSIL+ as a group within an HPV screening context. The overall PPV of hrHPV-positive, methylation-positive outcomes for ≥CIN2 in the study by Bonde *et al.* [18], was 36.7%, while in the current study, we report a PPV of 32.4%. This difference is presumably influenced by the variation in end-point terminology.

The specificity for HSIL+ using FAM19A4/miR124-2 methylation as a triage test in the current study is 68.2% (95% CI: 65.5–70.8). Results from Vink *et al.* [15] support that methylation analysis may be used to build robust triage algorithms with more objective stratification of women referred for colposcopy versus retesting compared with cytology, which the current study did not evaluate. Instead, we show that using genotyping with HONC and MONC together with FAM19A4/miR124-2 methylation leads to an increased specificity of 81.5% and could potentially be used instead of cytology. However, this approach leads to decreased sensitivity.

A limitation of the current study is the high rate of invalid methylation samples, 18%. Samples for the study were biobanked and kept at −80 degrees Celsius before DNA extraction. Samples consisted of residual material after the HPV APTIMA screening test and cytology analysis were performed. If a sample had few cells from the beginning, most material might have already been used in the screening. A triage method that yields many invalid results will impact screening suitability due to the need for retesting and resampling. More tests for methylation targets are on the market and could be validated and compared to current tests to find the best option for different settings.

Importantly, this study included women aged 30 years and older. A statistically significant difference was observed in the number of methylation-positive results between the 30–40 age group and the 60–70 age group, with a higher frequency of methylation positives in the older group. Previous studies have also shown that hypermethylation tends to increase with age [21,22]. Therefore, any molecular triage marker must be validated across all age groups to ensure its reliability and accuracy also in other studies and any molecular triage marker must be validated across all age groups.

A final approach using data from both cytology and molecular triage with genotyping and methylation resulted in the highest specificity, 91.3%, with a PPV of 60.2%. This result is higher than a previous study that showed that a combination of FAM19A4/miR124-2 methylation and cytology resulted in a specificity of 69.6% for CIN3 + , which did not include genotyping [15]. Including molecular triage tests along with cytology results, which was not the primary aim of the current study, has also been explored by a Dutch study group to predict clinical regression [23] of HSIL in histology with positive results. They reported that regression of dysplasia in women with a negative FAM19A4/miR124-2 methylation test compared with women with a positive test. The study also concluded that the regression was highest when HPV-16 was negative or when cytology showed ASCUS/LSIL (88.4%).

This study aimed to evaluate whether molecular triage of HPV-positive samples is as effective as cytology for cervical screening, with the goal of reducing the number of women requiring clinical examinations in cases where no dysplasia is present. Potentially, a combination of triage tests could aid in clinical decision-making but with an increased cost for performing additional testing methods. Any suggested molecular triage or combination of triage methods must also perform well on self-collected samples. In a setup where cytology is used as part of triage, this is not possible. Hence, in a future where self-sampling is increasingly implemented, as in Sweden, molecular options need to be further investigated.

## Conclusion

Based on the results of the methods evaluated in the current study, we do not propose a change to molecular triage in the current Swedish setting. This is due to either decreased sensitivity (hypermethylation) or decreased specificity (MONC/HONC genotyping) compared to cytology triage. Additional real-world screening studies, including other molecular triage assays, self-sampling, and all age groups, are needed to propose changes in the current screening algorithm.
